# Multi-Scale and Multi-Physics Models of the Transport of Therapeutic/Diagnostic Cancer Agents

**DOI:** 10.3390/cancers15245850

**Published:** 2023-12-15

**Authors:** Farshad Moradi Kashkooli, Michael C. Kolios

**Affiliations:** 1 Department of Physics, Toronto Metropolitan University, Toronto, ON M5B 1T8, Canada; 2 Institute for Biomedical Engineering, Science and Technology (iBEST), Keenan Research Centre for Biomedical Science, St. Michael’s Hospital, Toronto, ON M5B 1T8, Canada

## 1. Introduction

The effectiveness of tumor treatment heavily relies on the successful delivery of anticancer drugs. Solid tumors present physiological barriers, such as a dense extracellular matrix (ECM), enhanced solid stress and vessel compression, and irregularly shaped and leaky microvascular networks, reducing drug delivery efficacy [1,2,3]. These barriers and an absent or impaired lymphatic function contribute to elevated interstitial fluid pressure (IFP), hindering systematic drug delivery due to outward convection counteracting inward drug diffusion [1,2,3,4,5]. Each tumor possesses unique structural and physiochemical characteristics, such as the surface area of the vessel wall per unit volume for transport into the tissue, capillary network patterns, and hydraulic conductivity, making drug delivery and treatment highly complex and case-specific. The quantitative prediction of drug delivery to solid tumors using patient-specific data is crucial for clinical decision-making in therapeutic planning.

Mathematical modeling and simulation aim to enhance our understanding of tumor behaviors, ultimately improving treatment outcomes. Two major approaches for simulating drug delivery to solid tumors exist: the macroscopic and microscopic approaches. The macroscopic approach emphasizes the distribution of variables such as interstitial fluid velocity (IFV), IFP, and drug concentration over the tumor radius length scale. In the microscopic approach, characteristics like the microvascular network structure, blood flow within microvessels, and the interaction between blood flow and the microvascular wall are incorporated into the model. Given several transport processes involved in the delivery of therapeutic/diagnostic cancer agents and the complexity of the tumor microenvironment, sophisticated mathematical/computational modeling can be used to study the limitations of these approaches in treating/diagnosing cancer. Recently, multi-physics and multi-scale models have been applied to aid in the development of therapeutic/diagnostic agent delivery approaches. A wide range of mathematical models have been developed to mimic in vitro/in vivo/human biological and physiological environments and simulate these agents’ behaviors [6,7]. Various drug delivery systems (e.g., conventional chemotherapy [8,9,10,11,12,13,14], implantable systems [15,16], intraperitoneal chemotherapy [17,18,19,20], intratumoral injection [21], and anti-angiogenesis/vascular normalization combined with another therapy [22,23,24]) and diagnostic agents (e.g., the FDG PET radiotracer [25], the FMISO PET radiotracer [26], drug-loaded microbubbles [27], and radiopharmaceuticals [28]) have been explored using mathematical approaches.

On the other hand, medical imaging and image processing methods can be used to estimate, in principle, patient-specific variables that can be used to increase the accuracy of computational simulations. Consequently, there is considerable interest in predicting therapeutic/diagnostic agent delivery techniques through image-based computational modeling, facilitating personalized predictions. Different outputs of imaging modalities, particularly magnetic resonance imaging (MRI) [29,30,31], computed tomography (CT) [32], and diffusion tensor imaging (DTI) [33,34], can be used as inputs for these computational personalized models, enabling the timely and cost-effective optimization of drug delivery plans.

Nanoparticles have shown promise in preclinical studies for drug delivery in cancer treatment. Their drug-release kinetics and drug-loading capacity are crucial properties that determine treatment efficacy. In addition, stimulus-responsive nanoparticles, using external (magnetic, electric, light, laser, ultrasound, etc.) and/or internal (pH, redox, hypoxia, etc.) stimuli, significantly reduce the side effects of chemotherapeutics by controlling their release kinetics. Upon reaching the target site and responding to an internal or external stimulus, nanoparticles initiate drug release, either gradually (sustainable release) or rapidly (burst release), or as a combination of both [35,36]. However, nanoparticles encounter various barriers, such as rapid clearance and low transvascular rates. Some nanoparticles rely on the enhanced permeability and retention (EPR) effect for passive targeting, leading to their accumulation in tissues. Taking into account parameters associated with therapeutic agents (drugs and nanoparticles) and tumor-related factors, as well as the multiple scales involved (tissue, cell, and nano scales) and the physical nature of the transport process, computational modeling can be used to design efficient nano-sized drug delivery systems for solid tumors [6,37,38,39,40,41,42,43].

Computational models combine concepts and ideas from various disciplines, including pharmacokinetics, pharmacodynamics, fluid mechanics/dynamics, tissue mechanics, mass transport, heat transfer and biochemical processes. Multi-physics modeling allows for integrating information from various disciplines to inform the development of therapeutic/diagnostic agents [44]. Delivery processes can be evaluated separately or by combining all stages, aiding in identifying opportunities to maximize delivery outcomes and treatment effectiveness. This approach can reveal limitations in the transport and delivery mechanisms of the agents and can help guide therapeutic/diagnostic agents’ development. On the other hand, to improve the prediction capability of drug delivery, recent efforts in multiscale modeling to develop advanced models that can simulate transport processes across multiple length and time scales have emerged, integrating discrete and continuum modeling strategies [6,45]. These approaches promise to establish bottom-up in silico pipelines for drug design and delivery. Nevertheless, challenges persist in model parametrization and validation, particularly in the presence of variability introduced by various levels of heterogeneity in diseased tissues. When coupled with patient-specific data, these multi-physics and multi-scale models possess considerable potential for validation and, in some cases, personalization (such as creating digital twins) to facilitate their translation into routine clinical applications.

## 2. Highlights of the Special Issue

The current Special Issue aims to fill knowledge gaps, introduce a variety of mathematical and computational frameworks, and highlight the state-of-the-art research on multi-scale and multi-physics models in therapeutic/diagnostic agent development for cancer, aiming to demonstrate their potential clinical impact. In this Special Issue, original research articles and reviews are invited for submission. The topics covered include, but are not limited to, mathematical/computational modeling of delivery systems for theranostics, targeted delivery and nanomedicine more broadly.

Eight manuscripts were submitted for consideration in this Special Issue, and each underwent a rigorous review process by *Cancers*. Ultimately, five papers were accepted for publication and inclusion in this Special Issue. Concise descriptions of the published articles are listed below:

1. Mohammadi, M.; Sefidgar, M.; Aghanajafi, C.; Kohandel, M.; Soltani, M. Computational Multi-Scale Modeling of Drug Delivery into an Anti-Angiogenic Therapy-Treated Tumor. *Cancers* 2023, 15, 5464. https://doi.org/10.3390/cancers15225464
2. Rezaeian, M.; Heidari, H.; Raahemifar, K.; Soltani, M. Image-Based Modeling of Drug Delivery during Intraperitoneal Chemotherapy in a Heterogeneous Tumor Nodule. *Cancers* 2023, 15, 5069. https://doi.org/10.3390/cancers15205069
3. Hornsby, T.K.; Kashkooli, F.M.; Jakhmola, A.; Kolios, M.C.; Tavakkoli, J. Multiphysics Modeling of Low-Intensity Pulsed Ultrasound Induced Chemotherapeutic Drug Release from the Surface of Gold Nanoparticles. *Cancers* 2023, 15, 523. https://doi.org/10.3390/cancers15020523
4. Caddy, G.; Stebbing, J.; Wakefield, G.; Adair, M.; Xu, X.Y. Multiscale Modelling of Nanoparticle Distribution in a Realistic Tumour Geometry Following Local Injection. *Cancers* 2022, 14, 5729. https://doi.org/10.3390/cancers14235729
5. Bhandari, A.; Jaiswal, K.; Singh, A.; Zhan, W. Convection-Enhanced Delivery of Antiangiogenic Drugs and Liposomal Cytotoxic Drugs to Heterogeneous Brain Tumor for Combination Therapy. *Cancers* 2022, 14, 4177. https://doi.org/10.3390/cancers14174177

The contents of this Special Issue are outlined in Table 1, showcasing published papers spanning four distinct research domains: drug delivery, nano-sized drug delivery, drug release, and anti-angiogenesis. Table 1 provides in-depth insights into the diverse aspects of the physics, scales, and numerical approaches employed across these studies.

Contribution 1 introduces a sophisticated numerical model to investigate drug delivery with an anti-angiogenesis effect, surpassing previous research complexity. The model simulates intravascular and interstitial fluid flow, considering non-Newtonian blood behavior and a dynamically adapting microvascular network. The model evaluates solute exposure and uniformity in tumors of various sizes using a convection–diffusion–reaction model, incorporating anti-angiogenesis simulations. The findings demonstrate that anti-angiogenic therapy reduces drug wash-out in the tumor periphery. Notably, the microvascular structure and modifications induced by vascular normalization significantly impact drug delivery quality, showing a 39% improvement in uniformity with specific modifications in tumors with a radius of 0.2 cm.

Intraperitoneal (IP) chemotherapy holds promise for treating peritoneal carcinomatosis, yet limited drug penetration into tumors remains a challenge. Contribution 2 employs a numerical model incorporating an actual tumor image with heterogeneous vasculature to investigate IP chemotherapy. The tumor’s geometry is reconstructed using image processing techniques, and the model accounts for drug binding and uptake by cancer cells. After 60 min of IP treatment with doxorubicin (DOX), the area under the curve (AUC) for the average free drug concentration reaches 295.18 mol·m^−3^·s^−1^, and the half-width parameter W_1/2_, reflecting drug penetration, ranges from 0.11 to 0.14 mm. The treatment resulted in a fraction of killed cells reaching 20.4% by the end of the procedure. The findings emphasize the importance of considering specific vascular networks in modeling IP chemotherapy, showcasing the potential for patient-specific applications.

In contribution 3, a novel multiphysics simulation is developed to model LIPUS-induced doxorubicin (DOX) release from gold nanoparticle (GNP) drug carriers in an ex vivo tissue model. Transmission electronic microscopy imaging is performed before and after LIPUS exposure, and significant aggregation of the GNPs is observed upon DOX release. A numerical model is created to predict GNP aggregation and the subsequent DOX release via combining a thermal field simulation by solving the bioheat transfer equation (in COMSOL) and the Derjaguin, Landau, Verwey, and Overbeek (DLVO) total interaction potential (in MATLAB). The DLVO total interaction potential is found before and after LIPUS exposure, and an energy barrier for aggregation is determined. It is concluded that the interaction potential energy threshold for GNP aggregation (and, as a result, DOX release) is equal to 0.24 k_B_T. This simulation model can predict whether DOX release will occur for a series of GNP and LIPUS parameters. An extensive parametric study is also performed with the simulation model to study how GNP and LIPUS parameters affect DOX-loaded GNP colloidal stability. This model could optimize future ultrasound-triggered GNP-based drug delivery systems before clinical implementation.

Radiosensitizers enhance radiotherapy outcomes, and their effective distribution within the tumor is crucial due to their limited range. Contribution 4 introduces a computational model for nanoparticle transport within tumors, considering fluid velocity, particle deposition, and the convection–diffusion equation. Evaluating the impact of particle surface charge and injection locations reveals that negatively charged particles achieve a more uniform distribution, covering 100% of the tumor volume in the fluid and 44.5% of the deposited volume. Shifting the injection location towards the tumor’s center enhances the particle distribution. The findings suggest that negatively charged radiosensitizing particles and strategic injection sites can optimize their spread and penetration within the tumor.

Contribution 5 presents an image-based computational study of interstitial fluid flow and transport of liposomal cytotoxic and antiangiogenic drugs using an integrated approach that couples the key drug delivery processes in convection-enhanced delivery (CED) treatments. The novel contribution is the development of an integrated drug transport model to predict the dynamic changes of interstitial fluid flow and drug transport upon anti-angiogenesis. More importantly, the tumor’s 3D geometry and its heterogeneous characteristics, including its microvascular density, porosity, and cell density, are extracted from patient-specific dynamic MR images and applied as a model input to accommodate the realistic tumor properties. This study demonstrates that the co-infusion of antiangiogenic drugs can effectively improve cytotoxic drug delivery outcomes, facilitating enhanced drug penetration and accumulation. This can be attributed to the reduced microvascular density that inhibits the fluid exchange between the blood and tissue, mitigating cytotoxic drug dilution and drug loss due to blood drainage.

## 3. Conclusions and Future Research Need

Although submissions for this Special Issue are now closed, there is an ongoing need for sustained research and development in the realm of multi-scale and multi-physics models concerning the release, transport, and delivery of anticancer drugs (as well as diagnostic agents) to cancer. Considering the distinctive features of computational models, which include their widespread accessibility, cost-effectiveness, and the ability to integrate artificial intelligence, machine learning, and data inputs from imaging modalities, it is expected that multi-scale and multi-physics models for anticancer drug delivery will play a more prominent role in future cancer treatment strategies.

The primary hurdles in these models involve achieving a high resolution and accuracy in quantitative predictions while maintaining computational speed. Establishing a modeling framework and workflow that can concurrently address both objectives is imperative. The accuracy of these models is primarily dependent on parameterization related to the tumor, drug, nanoparticle, external source, and the physical models used to simulate the entire process. In practical terms, the intricacies of the drug delivery process require many model parameters, posing a distinct challenge in measuring all the parameter values. Validating computational models involving diverse physics introduces additional complexity. Simplifications and assumptions within the models are other factors that must be carefully verified before making final decisions.

## Figures and Tables

**Table 1:** Analysis of the published contributions in the Special Issue.

Contribution No./Research Area	Geometry Type	Different Physics Included	– Scale; – Software; – Numerical Approach	Main Outcomes
1/Drug delivery and anti-angiogenesis	Synthetic 2D geometry of tumor containing mathematically modeled microvascular network in a two-dimensional discrete space	Angiogenesis, anti-angiogenesis, intravascular blood flow, interstitial fluid flow, drug transport	– Multiscale (ranging from cell to tissue); – MATLAB and COMSOL; – An iterative computational method for modeling the intravascular blood flow in connection with the interstitial fluid flow and finite element method (FEM) for obtaining the concentration distribution in tissue.	This study emphasizes the crucial role of the capillary network, both quantitatively and qualitatively, underscoring its dual nature as a double-edged sword. Furthermore, it highlights the determinative impact of an anti-angiogenic agent in restructuring the microvascular network, a crucial factor for optimizing the quality of drug delivery.
2/Drug delivery	Two-dimensional tumor geometry extracted from vascularized tumor image	Interstitial fluid flow and drug transport	– Tissue; – COMSO; – FEM: The solution process comprises two distinct phases: the initial steady-state phase, which addresses the intravascular and interstitial fluid flow equations, and the subsequent time-dependent phase, dedicated to solving the mass transport equations. The outcomes of the steady-state solution serve as the foundational input for the subsequent transient simulations. Notably, the duration of the intraperitoneal (IP) treatment method necessitates that the transient simulations be executed within a one-hour timeframe.	The drug delivery during intraperitoneal (IP) chemotherapy is influenced by the specific vascular network of the tumor.
3/Ultrasound-triggered drug release from nanoparticles	Two-dimensional axisymmetric ex vivo tissue geometry with an embedded nanoparticle volume	Pressure acoustics (Helmholtz equation) and bioheat transfer (Penne’s bioheat transfer equation)	– Tissue; – MATLAB and COMSOL; – FEM and custom MATLAB code.	A numerical model framework is presented to predict drug release from nanoparticle drug carrier for any given ultrasound and nanoparticle parameters.
4/Nano-sized drug delivery	A realistic tumour geometry reconstructed from micro-CT images of an ex vivo FaDu tumour	The micro-scale model employs a particle trajectory tracking approach, meticulously computing the trajectories of individual particles in proximity to a cell. In contrast, the macro-scale model comprises two integral components: a nanofluid convection model and a nanoparticle transport model. The nanofluid convection model applies Brinkman’s equations to derive pressure and flow velocity, emphasizing the averaging of flow velocity across the representative elementary volume, not solely restricted to the fluid phase.	– Particle and tissue; – COMSOL; – FEM: The micro-model calculates particle deposition independently, while the macro-model simultaneously addresses interstitial fluid velocity and nanoparticle concentration. A linear scheme is predominantly employed for discretization, except in the velocity-solving phase, which utilizes a quadratic scheme. The models for particle tracing and nanofluid convection are resolved using the Generalized Minimal Residual Method (GMRES), while the nanoparticle transport model utilizes the Multifrontal Massively Parallel Sparse Direct Solver (MUMPS).	This study seeks to assess how the particle surface charge and injection site influence the distribution of nanoparticles, aiming to achieve an ideal and uniform concentration across the tumor. This research contributes to the advancement of radiosensitizer development and offers valuable insights for guiding clinical trials.
5/Nano-sized drug delivery and anti-angiogenesis	Three-dimensional realistic geometry of a brain tumor and its heterogeneous characteristics are extracted from the patient’s dynamic contrast-enhanced magnetic resonance (DCE-MR) images	Interstitial fluid flow, combined drug transport of cytotoxic and antiangiogenic drugs and antiangiogenic effects	– Tissue; – MATLAB and OpenFOAM; – For the extraction of patient-specific parameters from DCE-MRI, an in-house-developed MATLAB code iss used. For fluid flow and drug transport simulations, an in-house solver developed in OpenFOAM was used.	This study evaluates a novel treatment approach utilizing antiangiogenic and cytotoxic drugs, supported by mathematical modeling, revealing the potential benefits of this combined therapy in achieving a more uniform intratumoral environment and advancing the development of more effective brain tumor treatments.
